# Retroduodenal and Juxtapancreatic Schwannomas: Clinical and Surgical Insights From Three Cases

**DOI:** 10.7759/cureus.85916

**Published:** 2025-06-13

**Authors:** Akhil C Ganamani, Suresh K Palanichamy, Sankar Subramaniam

**Affiliations:** 1 Surgical Gastroenterology, Sri Ramachandra Institute of Higher Education and Research, Chennai, IND

**Keywords:** abdomen pain, immunohistochemistry staining, peripancreatic mass, retroperitoneal tumour, schwanomma

## Abstract

Retroperitoneal schwannomas, as such, are a rare entity, and those located near the pancreas and duodenum may emulate pancreatic tumors on imaging, making a diagnosis, surgical planning, and patient counselling challenging. Their low incidence and nonspecific clinical presentation often hinder preoperative identification.

This case series presents three female patients who presented with either mild epigastric discomfort or incidental findings during routine health evaluations. Contrast-enhanced CT scans revealed well-circumscribed, heterogeneous cystic lesions in the retroperitoneal space adjacent to the pancreas and duodenum. Each patient underwent a complete surgical excision of the tumor. Histopathological examination confirmed schwannomas, exhibiting typical Antoni A and Antoni B areas, as well as Verocay bodies. S-100 protein positivity was noted on immunohistochemical staining, supporting the diagnosis of a benign nerve sheath tumor. All patients experienced a smooth recovery after surgery, and no recurrences were noted during the follow-up period lasting for a year. These cases emphasise three different locations of retroperitoneal schwannomas: juxtapancreatic, retroduodenal, and posterior to the head of the pancreas, between the second part of the duodenum and the right kidney. A definitive diagnosis relies on histopathological and immunohistochemical findings. Surgical excision remains the preferred treatment and is associated with excellent outcomes.

## Introduction

Schwannomas are benign nerve sheath tumors arising from Schwann cells, which envelop peripheral nerves [1,2]. First described by Verocay in 1910, these mesenchymal neoplasms are composed exclusively of Schwann cells and lack neuroganglionic components [3]. Although schwannomas can occur throughout the body, their incidence within the abdominal cavity is uncommon. Retroperitoneal schwannomas, with a reported incidence of less than 3% of all schwannomas, demonstrate a greater tendency for spontaneous haemorrhage and degenerative changes compared to schwannomas arising in other anatomical regions [4,5]. Although the World Health Organization has excluded the term "malignant schwannoma" from its current classification, malignant transformation may occur, presenting as malignant peripheral nerve sheath tumors (MPNSTs) [6]. Accurate preoperative diagnosis is difficult, as these lesions are frequently mistaken for other retroperitoneal soft tissue neoplasms [7]. The use of biopsy in retroperitoneal Schwannomas remains somewhat debated. While CT-guided needle biopsy is feasible, potential risks include injury to adjacent organs, bleeding, infection, and tumor seeding [8]. Clinical presentation is often nonspecific, typically involving vague abdominal discomfort or incidental detection during imaging for unrelated conditions. Although imaging techniques like CT and MRI are helpful, retroperitoneal schwannomas often do not have specific features on scans. Certain nonspecific radiological signs may indicate schwannoma, such as solitary oval or sphere-shaped lesions. CT scans may pick up well-defined lesions with degenerative changes, including cysts, calcification, and necrosis, while MRI shows a T1 isosignal to skeletal muscle and a T2 hyperintensity or isosignal to skeletal muscle, with hypercellularity proportional to the T2 hyperintensity. Therefore, confirmation typically relies on histological and immunohistochemical testing after the tumor is surgically removed [9]. 

This case series presents three surgically treated cases of retroperitoneal schwannomas in three different retroperitoneal locations, aiming to highlight the diagnostic challenges and provide clinical and operative insights that may facilitate improved recognition and management of these rare tumors.

## Case presentation

Case one

**Figure 1:**
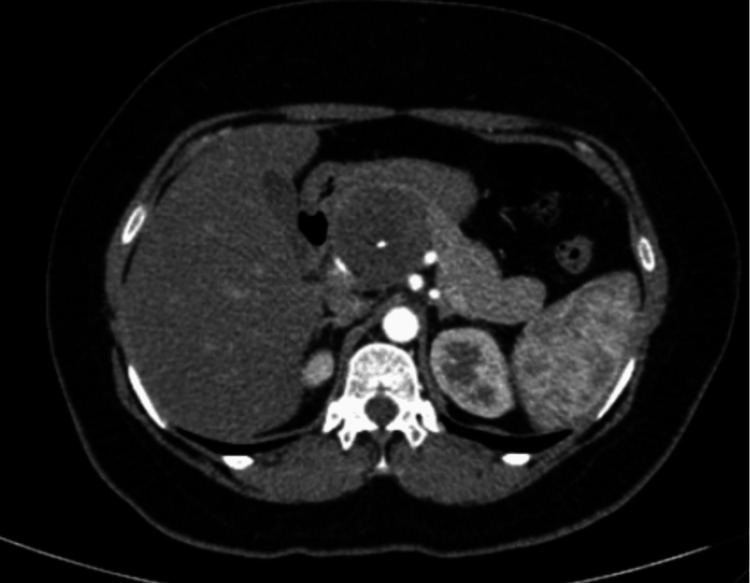
Arterial phase of CT scan showing the retroperitoneal tumor arising from the head of pancreas and blood vessels

**Figure 2:**
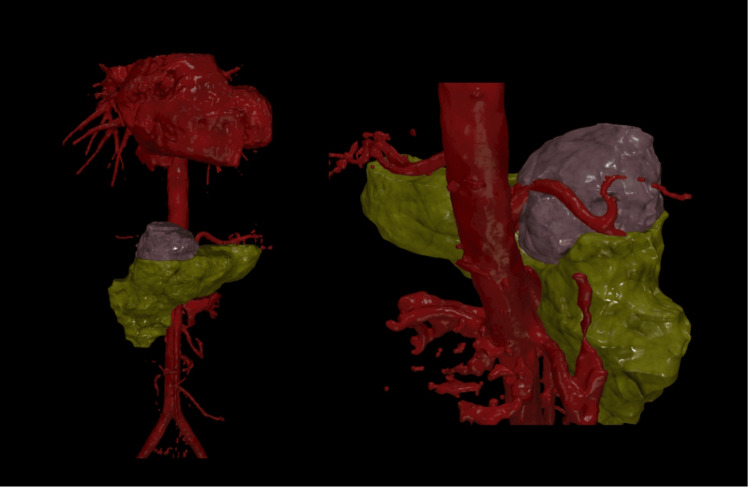
3D virtual reality reconstruction of tumour and pancreas and its association with arteries lilac color - tumour; green color - pancreas

**Figure 3:**
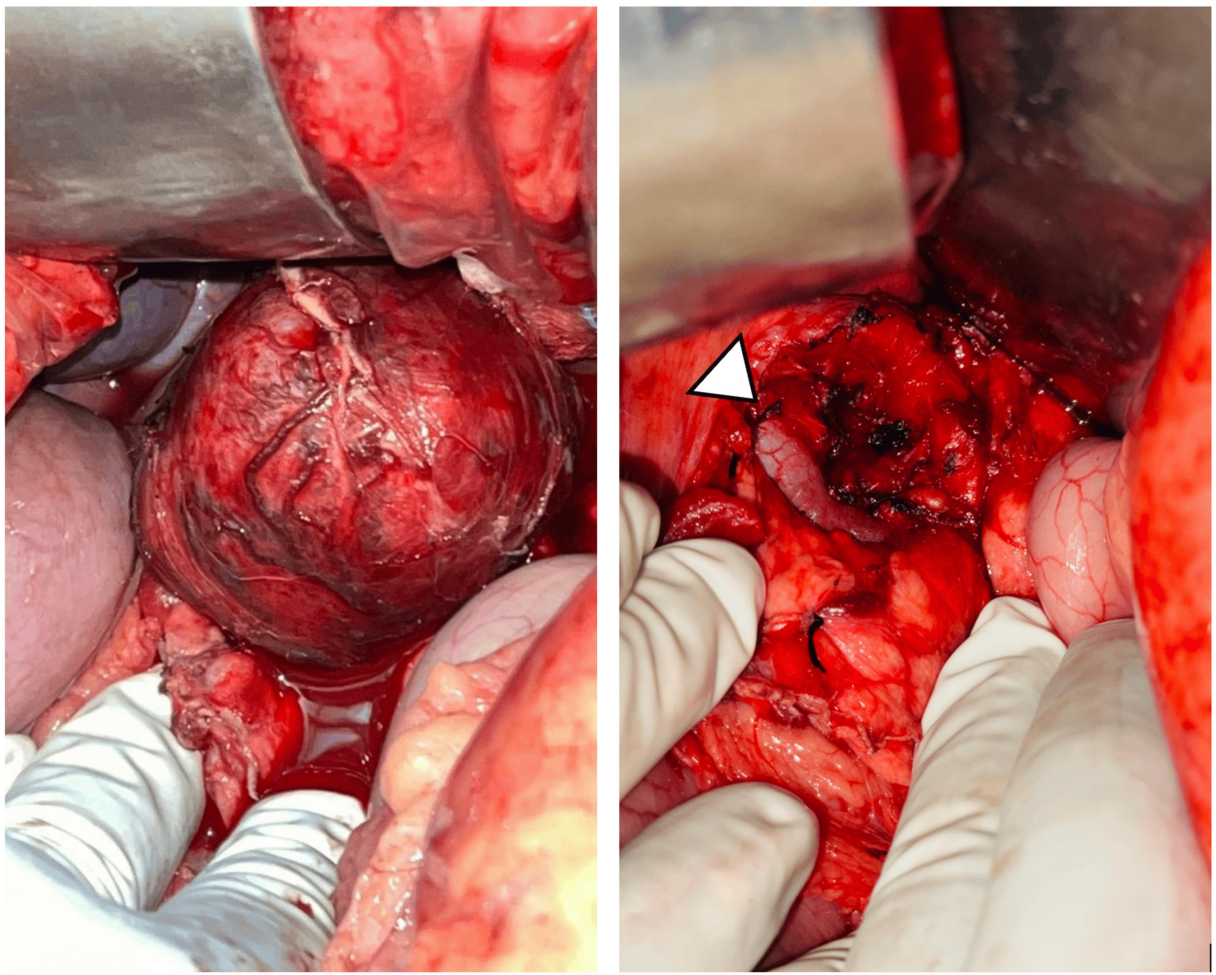
Intraoperative images A: On opening the lesser sac, a well-encapsulated tumor was noted at the superior border of the pancreas, and the tumor was splaying celiac axis branches; B: Post resection, the tumor bed showed its proximity to the celiac axis and its branch splenic artery (arrowhead).

**Figure 4:**
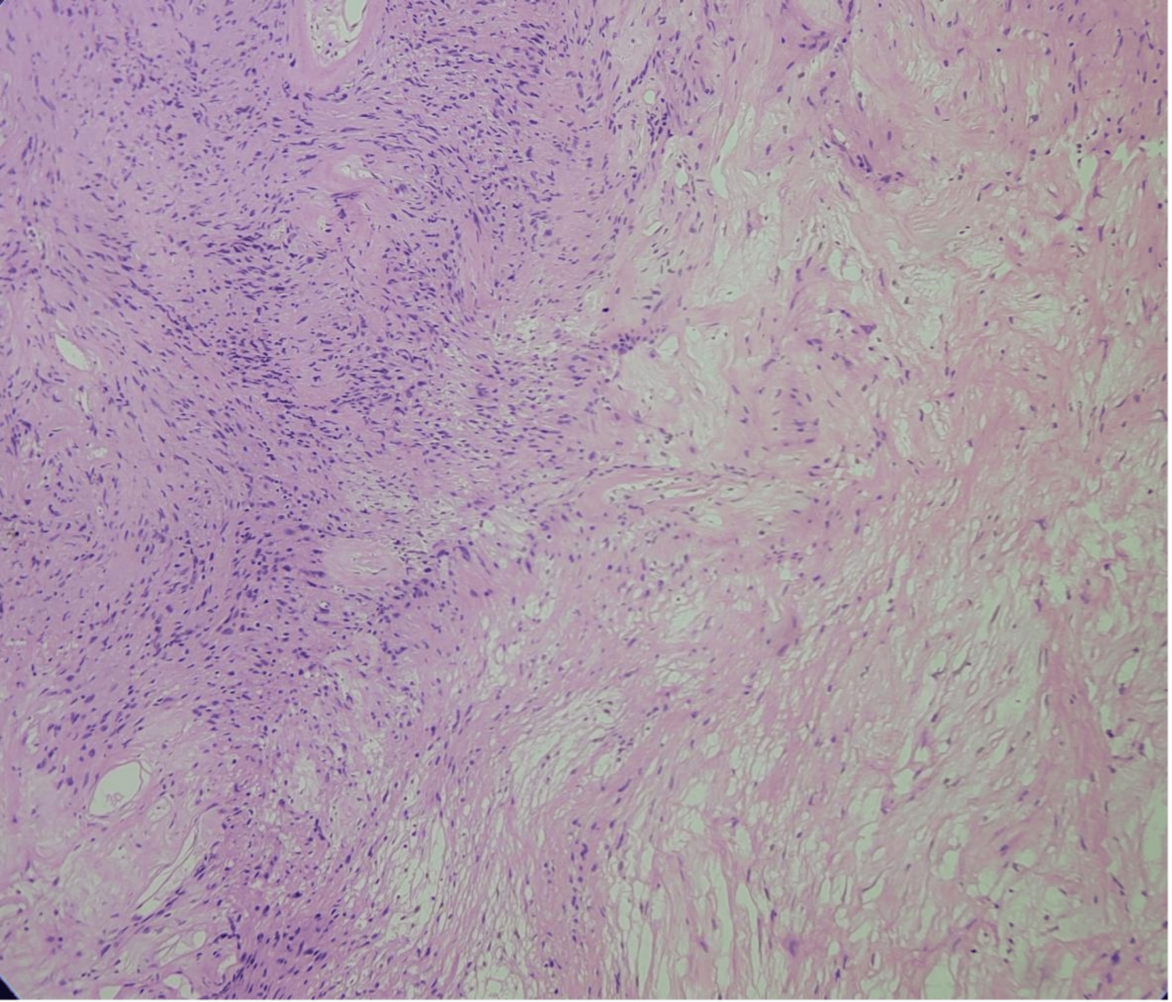
Spindle-shaped cells arranged in hyper and hypocellular areas

A 40-year-old woman with a history of hypothyroidism and an ECOG performance status of 1 came in with occasional upper abdominal (epigastric) pain that had been present for about a year. She described the pain as a dull, aching discomfort that sometimes radiated to her back. The physical examination was normal, and routine blood tests were within normal limits. An initial abdominal ultrasound found a cystic lesion near the head of the pancreas. A follow-up contrast-enhanced CT (CECT) scan showed a well-defined, multiloculated cystic mass of size 4.6 x 4.8 x 5.6 cm with foci of central calcification arising from the pancreatic head. The lesion was abutting the lesser curvature of the stomach and compressing the main portal vein and D2 segment of the duodenum. Posteriorly, the lesion was compressing the splenic artery (Figure 1). The images were 3D reconstructed in a virtual reality platform (Figure 2). 3D reconstruction helped us understand the relationship of blood vessels with respect to the tumor and aided us in predicting the location of the tumor. With a preoperative diagnosis of pancreatic head mass and tumor markers CA 19-9 and CEA being non-elevated, the patient was scheduled for a Whipple procedure. On exploring the lesser sac, the well-encapsulated tumor was noted to be juxta-pancreatic near the superior border of the pancreas and wedged between the common hepatic artery, splenic artery, splenic vein, portal vein, and neck of the pancreas. The tumor was excised using blunt and sharp dissection with the help of a harmonic scalpel, and pancreaticoduodenectomy was avoided (Figure 3). The postoperative period went smoothly, and her recovery was within the anticipated timelines. The histopathological examination revealed areas of hyper- and hypocellular composed of spindle-shaped cells, with immunohistochemistry showing strong positivity for S100 and Vimentin, which was suggestive of a schwannoma (Figure 4). She was followed up for a year and remained asymptomatic.

Case two

**Figure 5:**
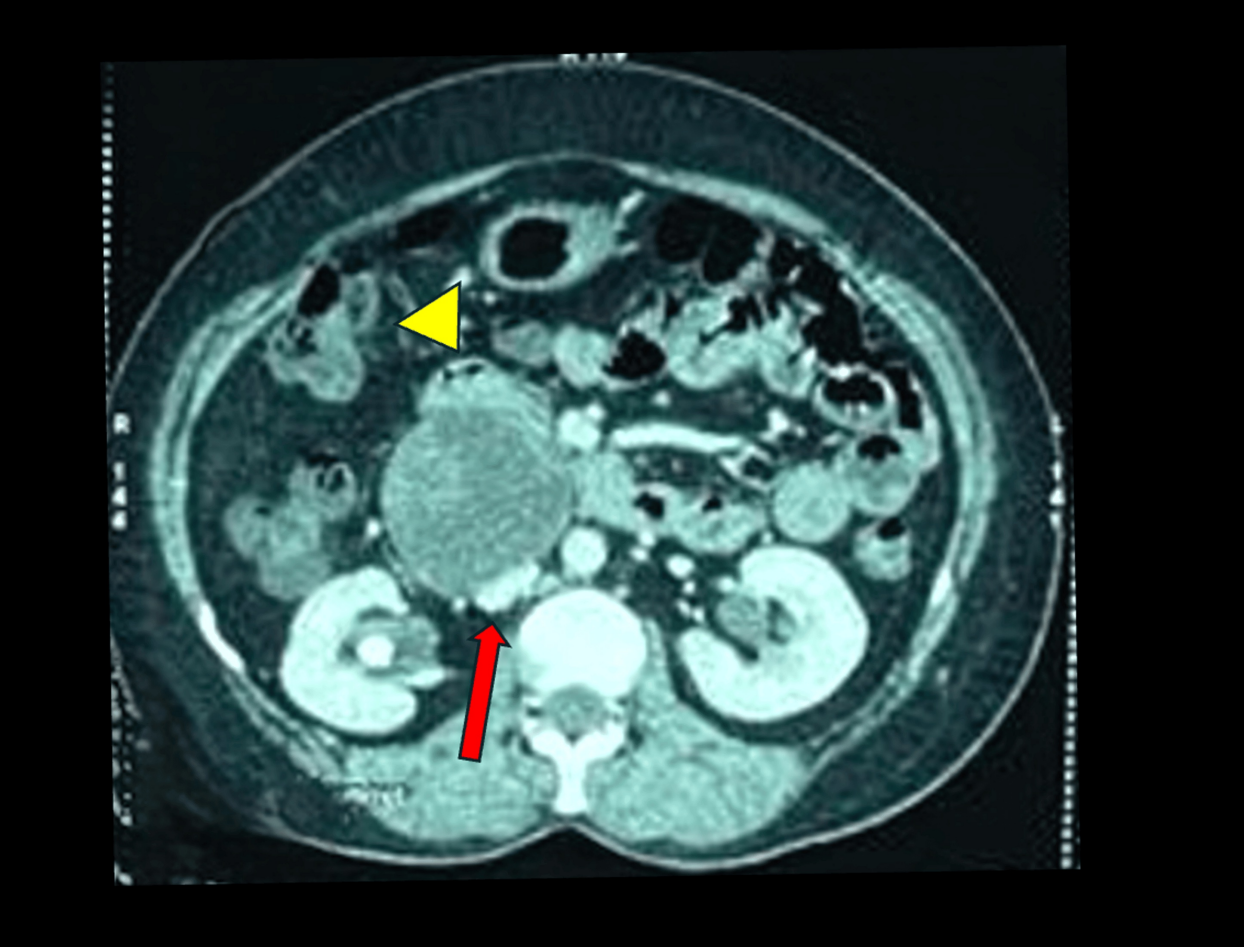
CT image showing the retroperitoneal heterogeneous cystic tumour and its relation to IVC and duodenum Red arrow - IVC, Yellow arrow head - duodenum IVC - inferior vena cava

A 38-year-old woman was referred for further evaluation after a retroperitoneal mass was unexpectedly discovered during a routine abdominal ultrasound. The mass measured about 4.7 x 5.5 x 7.5 cm and was reported to be arising from the head of the pancreas. She had no symptoms such as abdominal pain, weight loss, jaundice, or digestive problems. Her medical and surgical history was unremarkable, and she was not taking any regular medications. During the clinical evaluation, her condition remained stable. Physical examination of the abdomen was normal. Blood analysis, including tumor markers, was unremarkable. A contrast-enhanced CT scan of the abdomen revealed a tumor in the retroperitoneum posterior to the D2 segment of the duodenum and pancreatic head and in close proximity to the inferior vena cava (Figure 5).

**Figure 6:**
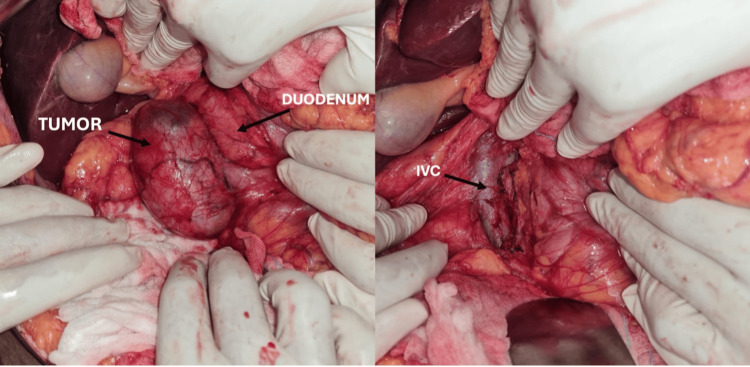
Intraoperative image A: After Kocherisation of the duodenum, an encapsulated cystic retroperitoneal tumor was noted posterior to the duodenum and anterior to the inferior vena cava (IVC). B: Tumor bed showing IVC after excising the tumour

**Figure 7:**
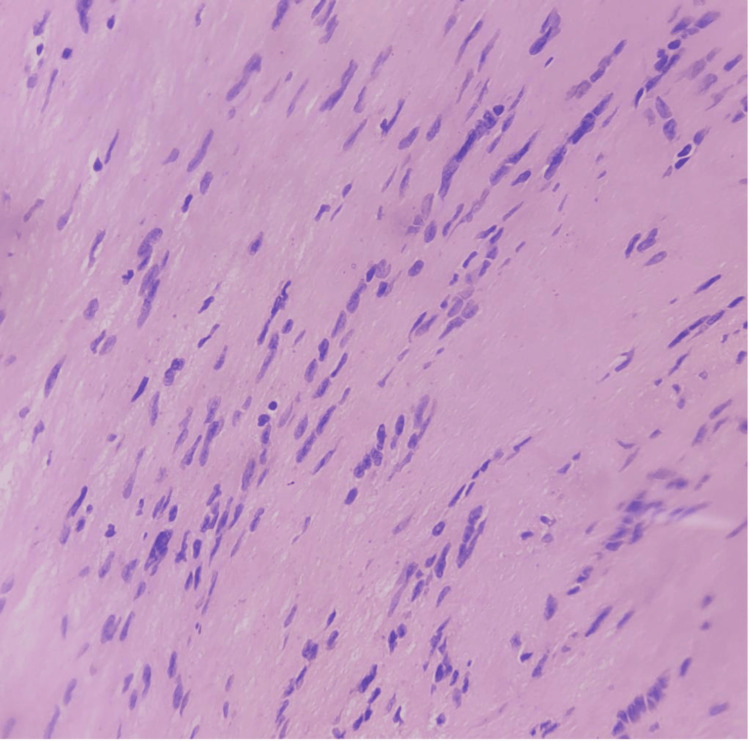
Verocay bodies Nuclear palisading with an anuclear zone in hypercellular areas make up Verocay bodies

The patient underwent exploratory laparotomy with a preoperative impression of a retroperitoneal tumor. Intraoperatively, a cystic encapsulated tumor of size 7 x 6 cm was identified in the retroduodenal and retropancreatic location, pushing the structures anteriorly and medially. Although adherent to the IVC, meticulous dissection enabled complete excision of the tumor (Figure 6). Histopathological examination confirmed the diagnosis of a benign schwannoma (Figure 7). The postoperative course was uneventful, and the patient remained asymptomatic during the follow-up period lasting for a year, with no evidence of recurrence on subsequent imaging.

Case three

**Figure 8:**
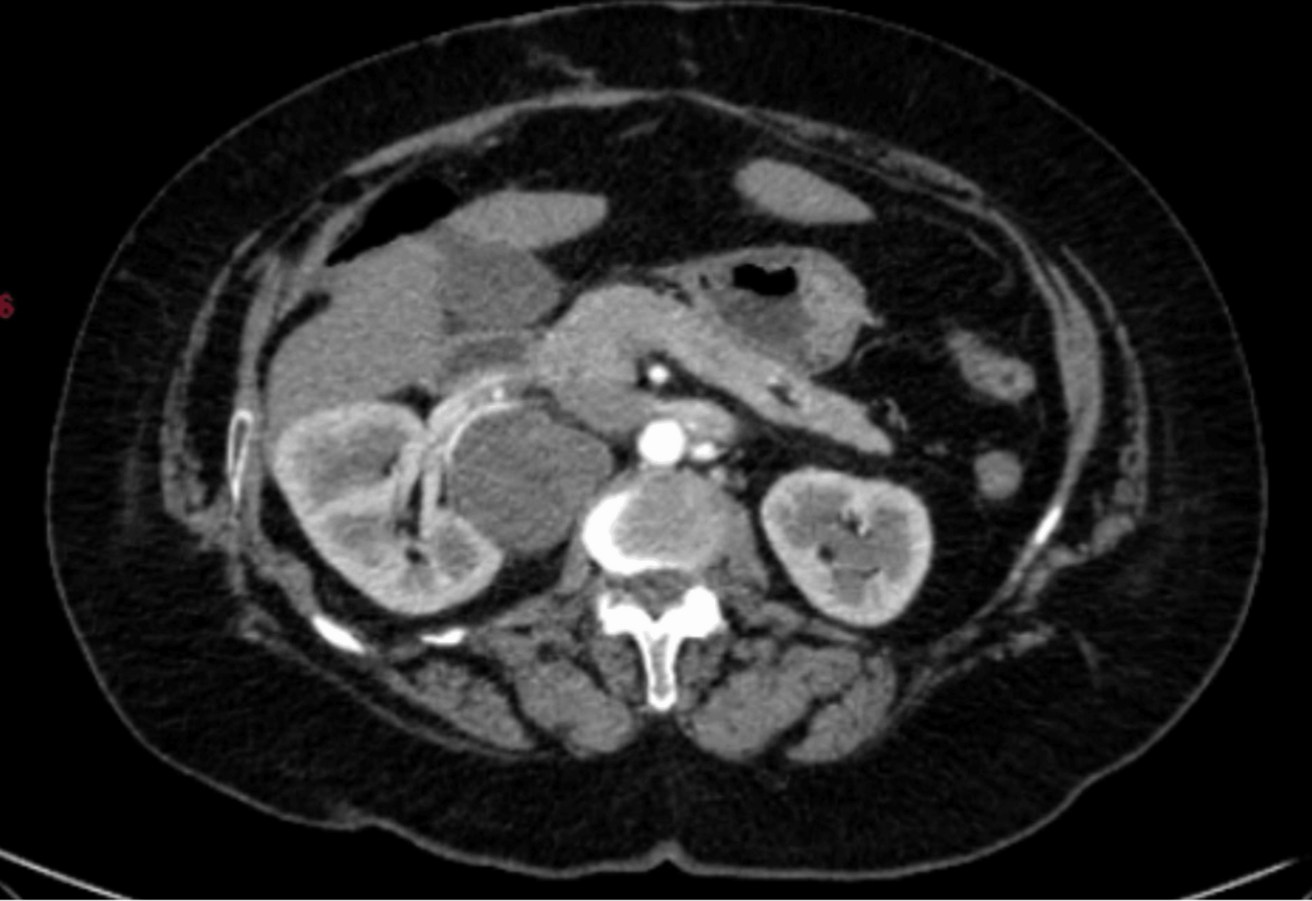
Axial section of the abdomen showing retroperitoneal lesion and its relation to kidney and duodenum

**Figure 9:**
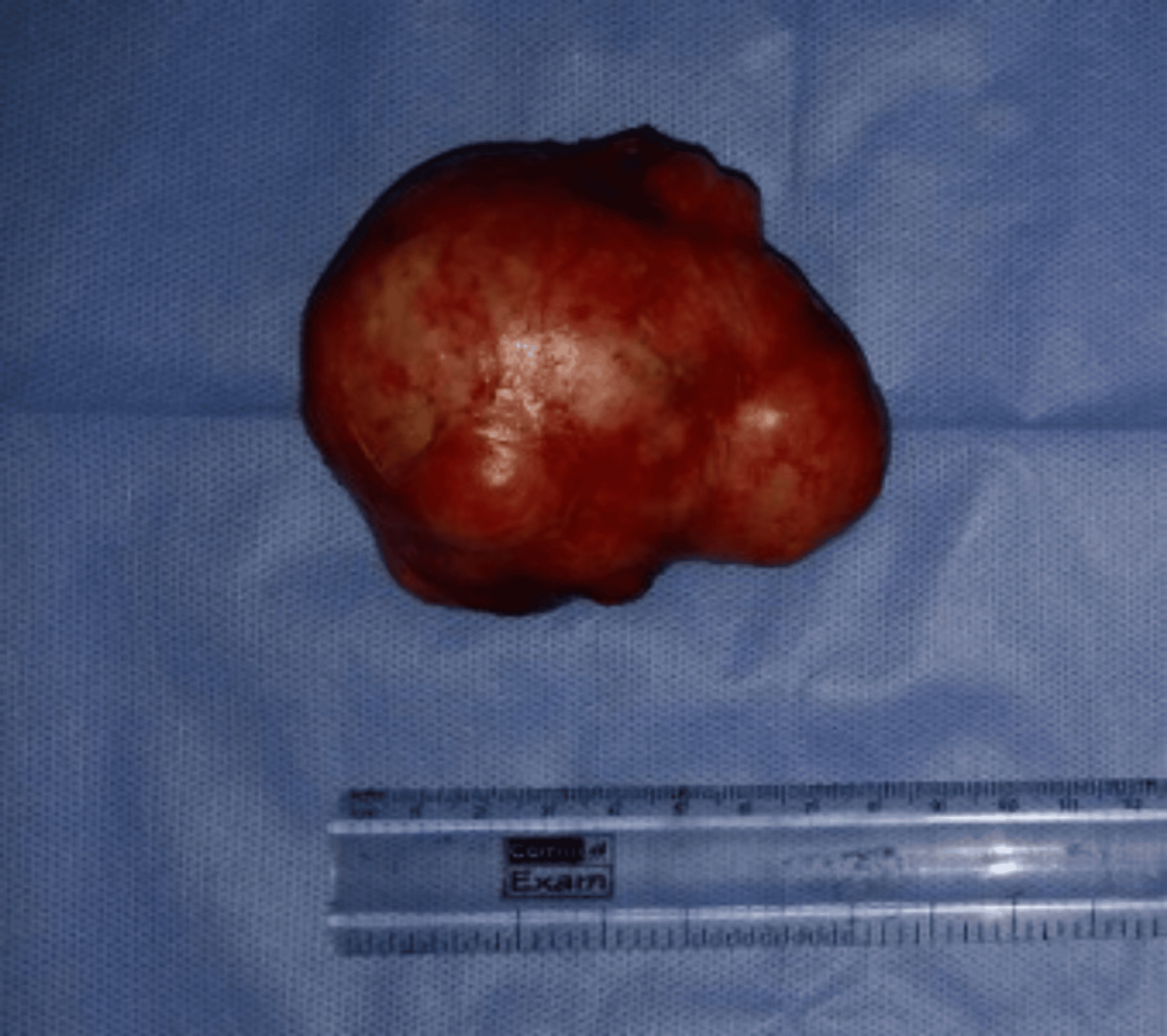
Operative specimen Excised specimen showing its encapsulated and lobulated surface with a reference scale

**Figure 10:**
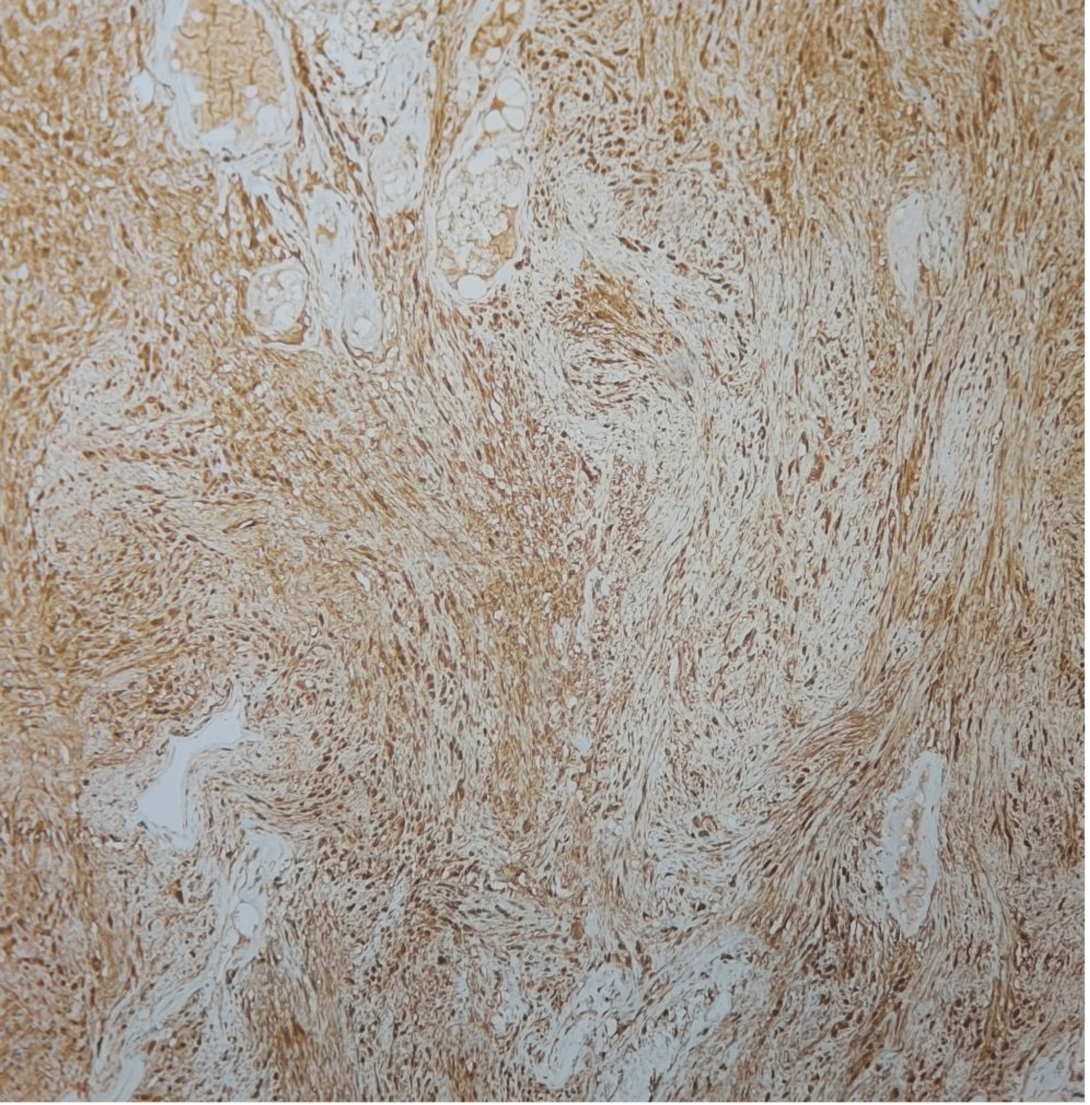
Immunohistochemistry showing S-100 positivity Diffuse staining suggestive of strong S-100 positivity which is diagnostic of Schwann cells

A 60-year-old female with a known history of diabetes mellitus presented with dull, aching epigastric pain, persisting intermittently over the previous three months. The pain radiated posteriorly toward the back, raising suspicion for a retroperitoneal origin. Her ECOG performance status was grade 1. Physical examination revealed no palpable abdominal mass, localized tenderness, or other notable findings. The abdomen was evaluated using contrast-enhanced computed tomography (CECT), which revealed a well-encapsulated heterogenous lesion of size 4.5 x 5.1 x 5 cm in the retroperitoneum, juxta renal aspect on the right side, with the kidney pushed posteriorly. The lesion is identified on the second-third part of the duodenum and posterior to the renal artery and vein (Figure 8). The patient underwent exploratory laparotomy wherein the encapsulated cystic retroperitoneal tumor was successfully excised after Kocherization of the duodenum and safeguarding the renal vessels (Figure 9). Histopathology evaluation of the tumor confirmed the diagnosis of schwannoma (Figure 10). The patient experienced an expected recovery and was followed up on for a year with no symptoms.

Table 1 summarizes the patient demographics.

**Table 1:** Summary of patient demographics, presenting complaints and cystic mass description

	Age (years)/Sex	Presenting complaints	Tumour size (cm)	CT description	Location of the tumor and its relation to the vessels
1	40 / Female	Intermittent epigastric pain radiating to the back for a year	4.6 x 4.8 x 5.6	Well-defined, multiloculated, heterogenous cystic mass with foci of calcification	Juxtapancreatic near the superior border of the pancreas. Wedged between the common heaptic artery, splenic artery and vein, portal vein, and neck of the pancreas
2	38 / Female	Asymptomatic	4.7 x 5.5 x 7.5	Well-defined, heterogenous cystic lesion	Retroduodenal and posterior to the head of the pancreas in close proximity to the inferior vena cava posteriorly.
3	60 / Female	Intermittent epigastric pain radiating to the back for 3 months	4.5 x 5.1 x 5 cm	Well-defined, heterogenous cystic lesion	Second part of the duodenum and the juxtarenal aspect posterior to the right renal vessels

## Discussion

Benign peripheral nerve sheath tumours originating from Schwann cells are termed schwannomas and also known as neurilemmomas. They are typically slow-growing and well-encapsulated, often discovered incidentally during imaging for unrelated conditions, as demonstrated in the second case of our series. Although they most commonly occur in the head, neck, and extremities, schwannomas located in the retroperitoneal or peripancreatic regions are exceedingly rare, accounting for fewer than 1% of all schwannomas [10].

In our case series, all three patients were middle-aged to elderly females with preserved functional status (ECOG 1). Two patients presented with nonspecific abdominal symptoms, such as dull epigastric discomfort, while one patient was entirely asymptomatic. These findings are consistent with the observations of Gubbay et al. [11], who reported that retroperitoneal schwannomas often remain asymptomatic until they grow large enough to compress adjacent structures.

Radiological imaging is critical in the preoperative evaluation of these tumours. In our series, contrast-enhanced computed tomography (CECT) revealed well-defined, encapsulated, heterogeneous cystic masses without evidence of invasion into adjacent organs. These imaging findings align with those reported by Hughes et al. [12], who noted that retroperitoneal schwannomas typically appear as hypodense or heterogeneously enhancing lesions on CT while preserving the surrounding fat planes. The absence of lymphadenopathy, vascular encasement, or distant metastasis in our patients further supports the benign nature of these tumours. Surgical excision is the preferred treatment option, offering an excellent prognosis and minimal risk of recurrence. [13]. In all three cases, despite proximity to major vascular structures such as the inferior vena cava, careful dissection enabled complete tumour removal without damage to adjacent organs or the need for vascular reconstruction (Table 1). Histopathological analysis of all excised specimens confirmed the diagnosis of schwannoma, revealing the characteristic biphasic architecture comprising Antoni A and Antoni B areas and the presence of Verocay bodies. Immunohistochemical staining showed strong positivity for S-100 protein, confirming the origin from Schwann cells, consistent with the findings of Das Gupta et al. [14]. Pancreatic schwannomas are especially rare and may radiologically mimic pancreatic tumours. The overlap in imaging characteristics and nonspecific clinical manifestations contributes to diagnostic ambiguity. Despite advances in imaging techniques, reliable preoperative identification remains elusive, and surgical resection is frequently required for definitive diagnosis. The limited number of reported cases further complicates the recognition of distinguishing radiologic and intraoperative features that may aid in differentiating these benign tumours from pancreatic malignancies [15].

Differentiating retroperitoneal schwannomas from other cystic or solid lesions in the peripancreatic and retroduodenal regions, such as cystic neoplasm of the pancreas, cystadenomas, or gastrointestinal stromal tumours (GISTs), can be challenging. However, the absence of invasive features, non-distinctive radiological findings, negative tumour markers (such as CA 19-9 and CEA), and the presence of a well-encapsulated lesion support a diagnosis of benign neurogenic neoplasm [16]. Schwannomas, when completely excised, have minimal to no risk of recurrence [17]. If left untreated, these tumors can grow significantly and lead to symptoms due to compression of surrounding structures.

## Conclusions

This case series highlights the anatomical variability and clinical implications of retroperitoneal schwannomas, which present at distinct sites, including the juxtapancreatic, retroduodenal, and juxtarenal regions. Despite differences in location, all tumours demonstrated similar radiological and pathological features, well-encapsulated, non-invasive lesions with strong S-100 positivity on immunohistochemistry. The proximity to vital structures such as the pancreas, duodenum, inferior vena cava, and renal vessels posed surgical challenges; however, complete excision was achieved in all cases without significant complications. These cases highlight that retroperitoneal schwannomas can mimic site-specific malignancies depending on their location and should be considered as a differential diagnosis. Awareness of their varied presentations is essential for avoiding overtreatment and for guiding appropriate surgical planning. Accurate imaging, careful intraoperative assessment, and definitive histopathological confirmation remain essential for the optimal management of these rare tumours.
